# Analysis of injuries and deaths from road traffic accidents in Iran: bivariate regression approach

**DOI:** 10.1186/s12873-022-00686-6

**Published:** 2022-07-18

**Authors:** Soodeh Shahsavari, Ali Mohammadi, Shayan Mostafaei, Ehsan Zereshki, Seyyed Mohammad Tabatabaei, Mohsen Zhaleh, Meisam Shahsavari, Frouzan Zeini

**Affiliations:** 1 Department of Health Information Technology, Faculty of Allied Sciences, Kermanshah University of Medical Sciences, Kermanshah, Iran; 2 Department of Biostatistics, Faculty of Health, Kermanshah University of Medical Sciences, Kermanshah, Iran; 3 Inflammation Research Center, Tehran University of Medical Sciences, Tehran, Iran; 4 Department of Medical Informatics, Faculty of Medicine, Mashhad University of Medical Sciences, Mashhad, Iran; 5 Department of Anatomy and Cell Biology, Medicine Faculty, Kermanshah University of Medical Sciences, Kermanshah, Iran; 6 Imam Ali Hospital Heart Center, Kermanshah University of Medical Sciences, Kermanshah, Iran

**Keywords:** Road Traffic Accident, Death, Injury, Bivariate Regression

## Abstract

**Backgrounds:**

This study aims to estimate and compare the parameters of some univariate and bivariate count models to identify the factors affecting the number of mortality and the number of injured in road accidents.

**Methods:**

The accident data used in this study are related to Kermanshah province in march2020 to march2021. Accidents areas were divided into 125 areas based on density characteristics. In a one-year period, 3090 accidents happened on the suburban roads of Kermanshah province, which resulted in 398 deaths and 4805 injuries. Accident information, including longitude and latitude of accident location, type of accident (fatal and injury), number of deaths, number of injuries, accident type, the reason of the accident, and the kind of accident were all included as population-level variables in the regression models. We investigated four frequently used bivariate count regression models for accident data in the literature.

**Results:**

In bivariate analysis, except for the DNM model, there is a reasonable decrease in the AIC measures of the saturated model compared to the reduced model for the other three models. For the injury models, MSE is lowest, respectively for DIBP (137.87), BNB (289.46), BP (412.36) and DNM (3640.89) models. These results are also established for death models. But, in univariate analysis, only injury models almost present reasonable results.

**Conclusions:**

Our findings show that the IDBP model is better suitable for evaluating accident datasets than other models. Motorcycle accidents, pedestrian accidents, left turn deviance, and dangerous speeding were all significant variables in the IDBP death model, and these parameters were linked to accident mortality.

**Supplementary Information:**

The online version contains supplementary material available at 10.1186/s12873-022-00686-6.

## Backgrounds

Today, one of the biggest challenges in the world, especially in developing countries, is traffic accidents and their consequences. Road accidents include a variety of elements and conditions. Some of this data is geographically specific, while others are descriptive of accidents [1], which are caused by driver behavior, road characteristics, and environmental variables [2–5]. Also, road accidents are one of the most serious public health issues in the world [6], with an estimated 1.2 million people killed and 50 million people wounded each year [7]. According to forensic statistics in Iran, the average traffic fatality over the past ten years has been 22,185 individuals [8]. Moreover, about 70% of accidents in this country have occurred on roads and suburban roads, and usually, the victims are healthy young people, and about 10% of them died in accidents [9]. According to United Nations data, 33 people have perished in traffic accidents for every 10,000 automobiles [10]. Accident-related fatalities and injuries have been recognized as a worldwide problem, and traffic safety concerns have been a significant concern since the dawn of the vehicle age, over a century ago [11]. The cost of fatalities and injuries from traffic accidents has a significant impact on society. In recent years, researchers have paid more attention to determining the factors affecting the severity of driver injuries caused by traffic accidents [12–14]. The number of traffic accidents and their effects, especially casualties, justify the importance of analyzing the factors affecting their occurrence [15–17].

Traffic control and research centers typically use descriptive statistical techniques and graphs, such as frequency tables, bar charts, and histograms to organize the number of casualties in road accidents. This statistical approach provides only a descriptive report of the rate of accidents, and based on this information, no estimate and prediction can be made. It goes without saying that having a good model for predicting and evaluating these implications is critical. It's also crucial to figure out how persons, the environment, road conditions, and vehicle types affect traffic accidents. In such analyzes, the response variable measurements are counted and the important issue is whether the counting data have a normal distribution in terms of their quantitative scale, and whether these data can be modeled using linear regression? Various studies have shown that counting variables meet the following conditions: they have a counting nature, i.e. there are no negative numbers and the amplitude of data changes starts from zero. They're also often skewed, thus they don't have a normal distribution and may be sparsely dispersed. As a result, these variables fail to fulfill the criteria for linear regression [18]. Hence, for better description and modeling, it is necessary to use the nonlinear count regression models are used, such as Poisson regression, negative binomial regression, and quasi Poisson regression [18, 19]. Although the distribution of univariate discrete data is well developed, there may be more than one response variable in some studies, such as traffic accidents analysis [20, 21]. On the other hand, road accidents have consequences such as death and injury, and the analysis of the simultaneous of these two responses has been rarely used in studies. Because the marginal distributions are not independent, it is occasionally required to utilize a bivariate distribution for correlated bivariate data.

In recent years, the multivariate model with two or more replies and counting data has gotten a lot of attention [22]. In fact, in addition to univariate models, bivariate regression models have been proposed for the analysis of two correlated response variables. These models provide sufficient flexibility by allowing two correlated response variables that different predictors influence. Besides, a bivariate model is more useful for inference and prediction purposes because it allows us to correctly determine the dependencies between two dependent variables [23]. In recent years, different models were developed that each has some advantages and disadvantages and may present different results in different situations [24]. The bivariate count regression has shown to be challenging, because of correlation structure is not a specified parametric class of distributions.

The bivariate Poisson model is the most extensively used, although it has the same constraints as the univariate Poisson model, and the variance estimates of the model are influenced when there is over-dispersion or under-dispersion in the data. Furthermore, the presence of negative correlation and heterogeneity in variance decreases the model's efficiency [23]. Bivariate negative binomial (BNN) regression, Dirichlet negative multinomial (DNM) regression model, and diagonal inflated bivariate Poisson (DIBP) regression are introduced in the literature for the solution of these issues and have better performance to describe bivariate responses that are interdependent and also have the feature of hyper diffraction [25, 26]. Therefore, this study aimed to estimate and compare the parameters of these models to identify the factors affecting the number of mortality and the number of injured in road accidents.

### Some previous studies to analyzing accident data

Over the years, a number of count regression models have been used to analyze accident data. The univariate Poisson regression and negative binomial models were utilized as a starting point for the majority of the models employed in the area. For example, in 2018, Mandacar and colleagues [27] conducted research on traffic-related deaths and severe injuries in Goiânia, Brazil. They used Poisson regression to fit the data, and then univariate analysis was used to analyze it. Moreover, recently Sagamiko and Mbare in 2021 implemented study for Modelling Road Traffic Accidents Counts in Tanzania. They used univariate Poisson regression for injuries and death accidents and ignored correlation between them [28].

In fact, accident variables, including to different levels, such as injuries and fatalities that are usually affected by big correlations and analyses based on separate models are inappropriate, and bivariate models are needed. Unfortunately, the majority of research in the area of accidents has utilized models that aren't specifically created for multivariate or bivariate models, instead considering the correlation of variables using standard models with modifications. Table 1 shows an overview of some of the research that looked at this topic.

**Table 1:** Summary of some previous studies to analyzing accident data

Model type	Year	Conclusion
A joint model with Weighted risk score to combine crash count and crash severity [29]	2020	using of crash severity and crash count amended the accuracy of prediction model
A bivariate Bayesian hierarchical extreme value model for traffic conflict-based crash estimation [30]	2020	The bivariate model estimate regression coefficients more precisely than univariate models
Bayesian multivariate hierarchical spatial joint model [31]	2018	This model has a better fit for the crash data compared to the univariate alternative model
Copula-Based Joint Model of Injury Severity and Vehicle Damage in Two-Vehicle Crashes [32]	2015	On the basis of goodness-of-fit statistics, the Gaussian copula model that was calculated interrelationships between injury severity and vehicle damage was suitable
using the random-parameters tobit model for factors affecting highway accident rates [33]	2012	The empirical results show that this model was proper fit to the data
A joint-probability approach to crash prediction models [34]	2011	Joint probability model that modeled Crash occurrence and severity simultaneously, shown the good fit for data
Multivariate Poisson-Lognormal Models for Jointly Modeling Crash Frequency by Severity [35]	2007	The results show multivariate model that accounted correlation of variables, was achieved more accurate estimates

### Data sources and study population

**Fig. 1:**
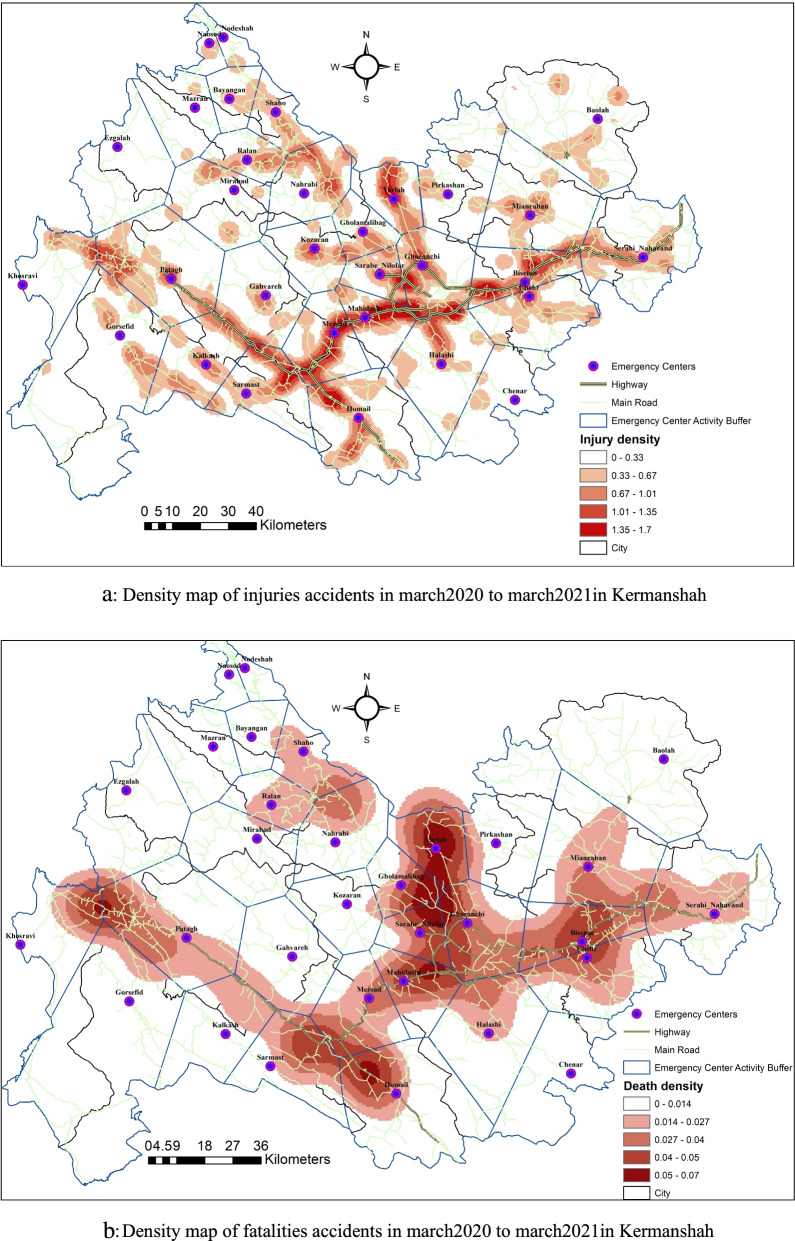
**a** Density map of injuries accidents in march2020 to march2021in Kermanshah. **b** Density map of fatalities accidents in march2020 to march2021in Kermanshah

The accident data used in this study are related to the research area of ​​Kermanshah province. Kermanshah province, with an area of ​​25,009 square kilometers and a population of 1,945,227 people, is located at latitude 34.308159 and longitude 4,705,732, and the geographical position is 34.308159° north and 47.05732° east in western Iran. The Kermanshah province's Traffic Accident Command and Control Center provided these figures. The characteristics of accidents are shown in Fig. 1. The majority of accidents, as seen in these graphs, have happened on major highways. The roads from Kangavar to Qasr Shirin on the Karbala highway, as well as the route from Kamyaran to Kermanshah, were the most accident-prone.

### Outcome variables

In a one-year period, 3090 accidents happened on the suburban roads of Kermanshah province, which resulted in 398 deaths and 4805 injuries. Figure 1a shows the density map of injury accidents. The highest density of injury accidents was in the axes of Mahidasht to Sarmast, Kamyaran axis, and Sarpol-e-Zahab-Qasr-e-Shirin axis. 1.36 to 1.7 people per square meter of injuries occurred in these places. Figure 1b shows the density map of fatal crashes. The highest density of fatal accidents was in Mahidasht axis, Kamyaran axis, Sarpol-e-Zahab and Qasr-e-Shirin axis, Hamil axis, and Sarab Niloufar axis. 0.28 to 0.35 people per square meter of fatal accidents occurred in these places.

### Predictors

Population-level variables that were included in the regression models were accident information, including:

Date, time and place of accident, longitude, and latitude of accident place, the crash location latitude and longitude coordinates, obtained by GPS or mapping type of accident (fatal and injury), number of fatalities, number of injuries, accident mode (multiple vehicles (Collisions between motor vehicles), off-road (Off-road motor vehicle), fixed object (Collisions between a motor vehicle and fixed object), pedestrian (Collisions between a motor vehicle and pedestrian), Collisions between a motor vehicle and motorcycle, Overturning (Overturning a motor vehicle), etc.), the cause of the accident (deviation to the left, speeding (high speed motor vehicle), reversing (Vehicle rotation), fatigue and drowsiness (Drivers who don’t get enough sleep), lack of attention of driver to the front, non-compliance with the right of way, non-compliance with the longitudinal and transverse distance, technical defects, etc.), and the type of vehicle (freight, personal ride, public passenger and motorcycle).

### Software

Data were analyzed using ArcGIS 10.2 and R4.0.5 software. The R packages to be used included ggplot2, msme, Count, moments, Mass, MGLM, BPglm and bivpois. Significant level in this study is considered at 0.05.

### Statistical analysis

In recent years, joint modeling of two or more counting outcomes has gotten a lot of interest. When two counting variables are associated and need to be estimated together, bivariate counting models may be utilized. Various models have been devised, each with its own set of benefits and drawbacks that may provide different outcomes in different scenarios. We have compared univariate Poisson, negative binomial regression and the four bivariate count regression models for accident data used commonly, which summary of them are mentioned below and reader can refer to their references for more details.

### Poisson regression

In statistics and probability, the Poisson process is used to represent a sequence of events with discrete values under the following conditions, and the random variable in this process has a Poisson distribution.

1. The number of event occurs in a specific time or place interval.
2. The probability of each event occurring is independent of the occurrence of other events, that is, the events are independent of each other.
3. The average number of events per time period is constant.
4. It is not possible for two events to occur at the same time.

A discrete random variable X is said to be a Poisson random variable with parameter $\uplambda$ is shown as $\mathrm{X}\sim \mathrm{p}(\uplambda )$ that

$$\mathrm{p}\left(\mathrm{X}=\mathrm{x}\right)=\frac{{\mathrm{e}}^{-\uplambda }{\uplambda }^{\mathrm{x}}}{\mathrm{x}!};\mathrm{ x}=\mathrm{0,1},\dots ,\infty ,\uplambda >0$$

$$\mathrm{E}\left(\mathrm{x}\right)=\mathrm{Var}\left(\mathrm{x}\right)=\uplambda$$

Almost all numerical models have the basic structure of a linear model, and in Poisson regression, the difference is that the equation on the left is expressed as a logarithm:

$$\mathrm{ln}\left(\uplambda \right)={\upbeta }_{0}+{\upbeta }_{1}{\mathrm{X}}_{1}+{\upbeta }_{2}{\mathrm{X}}_{2}+\dots +{\upbeta }_{\mathrm{k}}{\mathrm{X}}_{\mathrm{k}}$$

Note that there is no linear relationship between the model and predictive (independent) variables such as the linear model, and that there is a linear relationship between the natural logarithm of $\uplambda$ and the predictor variables. An important feature of the natural logarithm link function for counting data is that it ensures that predicted values will always be positive. A process called maximum likelihood estimation (MLE) may be used to estimate β values.

### Bivariate poisson regression

The bivariate Poisson regression models are the most widely used for bivariate counts data. These models [36] define the correlation structure through the trivariate reduction method, where the pair of dependent variables is specified using three random variables. Consider a pair of random variables (X, Y) that have a common distribution as follows:

$$\mathrm{P}(\mathrm{X}=0,\mathrm{ Y}=0) ={\mathrm{p}}_{00} ,\mathrm{ P}(\mathrm{X}=1,\mathrm{ Y}=0) ={\mathrm{p}}_{10} ,\mathrm{ P}(\mathrm{X}=0,\mathrm{ Y}=1) ={\mathrm{p}}_{01} ,\mathrm{ P}(\mathrm{X}=1,\mathrm{Y}=1) ={\mathrm{p}}_{11}$$

that $${\mathrm{p}}_{00}+{\mathrm{p}}_{10}+{\mathrm{p}}_{01}+{\mathrm{p}}_{11}=1$$

First, bivariate Poisson distribution is constructed that the probabilities are denoted as ${\mathrm{p}}_{11}=\frac{{\uplambda }_{11}}{\mathrm{n}}, {\mathrm{p}}_{01}=\frac{{\uplambda }_{01}}{\mathrm{n}}, {\mathrm{p}}_{10}=\frac{{\uplambda }_{10}}{\mathrm{n}}$ then joint distribution for n independent vectors (${\mathrm{X}}_{1},{\mathrm{Y}}_{1}),\dots ,\left({\mathrm{X}}_{\mathrm{n}},{\mathrm{Y}}_{\mathrm{n}}\right)$ is defined $\mathrm{P}(\sum_{\mathrm{l}=1}^{\mathrm{n}}{\mathrm{X}}_{\mathrm{l}}=\mathrm{k},\sum_{\mathrm{l}=1}^{\mathrm{n}}{\mathrm{Y}}_{\mathrm{l}}=\mathrm{l})=\sum_{\updelta =\mathrm{max}\left(\mathrm{k}+\mathrm{l}-\mathrm{n},0\right)}^{\mathrm{min}\left(\mathrm{k},\mathrm{l}\right)}\frac{\mathrm{n}!}{(\mathrm{n}-\left(\mathrm{k}+\mathrm{l}\right)+\updelta )!(\mathrm{k}-\updelta )!(\mathrm{l}-\updelta )!\updelta !}={ \left(1-\frac{{\uplambda }_{10}+{\uplambda }_{01}+{\uplambda }_{11}}{\mathrm{n}}\right)}^{\mathrm{n}-\left(\mathrm{k}+\mathrm{l}\right)+\updelta }{\left(\frac{{\uplambda }_{10}}{\mathrm{n}}\right)}^{\mathrm{k}-\updelta }{\left(\frac{{\uplambda }_{01}}{\mathrm{n}}\right)}^{\mathrm{l}-\updelta }{\left(\frac{{\uplambda }_{11}}{\mathrm{n}}\right)}^{\updelta }$

In the above equation, the right side converges to the following equation:

$$\frac{{{\uplambda }_{10}}^{\mathrm{k}-\updelta } {{\uplambda }_{01}}^{\mathrm{l}-\updelta } {{\uplambda }_{11}}^{\updelta }}{\left(\mathrm{k}-\updelta \right)!\left(\mathrm{l}-\updelta \right)!\updelta !} {\mathrm{e}}^{-({\uplambda }_{10}+{\uplambda }_{01}+{\uplambda }_{11})}$$

Then the partial distribution of the sum vector (X, Y) is as follows which gives the marginal distribution of BP distribution:

$$\mathrm{P}(\mathrm{X}=\mathrm{k },\mathrm{Y}=\mathrm{l})=\sum_{\updelta =0}^{\mathrm{min}(\mathrm{k},\mathrm{l})}\frac{{{\uplambda }_{10}}^{\mathrm{k}-\updelta } {{\uplambda }_{01}}^{\mathrm{l}-\updelta } {{\uplambda }_{11}}^{\updelta }}{\left(\mathrm{k}-\updelta \right)!\left(\mathrm{l}-\updelta \right)!\updelta !} {\mathrm{e}}^{-({\uplambda }_{10}+{\uplambda }_{01}+{\uplambda }_{11})}$$

If $\left({\mathrm{X}}_{\mathrm{i}},{\mathrm{Y}}_{\mathrm{i}}\right)\sim \mathrm{BP}\left({\uplambda }_{1\mathrm{i}},{\uplambda }_{2\mathrm{i}},{\uplambda }_{3\mathrm{i}}\right)$ then BP regression model is defined as following that ${\mathrm{w}}_{\mathrm{ki}}$ is the vector of ith predictor and ${\upbeta }_{\mathrm{k}}$ is the vector of kth regression coefficient.

$$\mathrm{log}({\uplambda }_{1\mathrm{i}})= {\mathrm{w}}_{1\mathrm{i}}\ {~}_{\top} \ {\upbeta }_{1} ,$$

$$\mathrm{log}({\uplambda }_{2\mathrm{i}})= {\mathrm{w}}_{2\mathrm{i}}\ {~}_{\top} \ {\upbeta }_{2} ,$$

$$\mathrm{log}({\uplambda }_{3\mathrm{i}})= {\mathrm{w}}_{3\mathrm{i}}\ {~}_{\top} \ {\upbeta }_{3}$$

### Bivariate negative binomial regression

Based on a similar correlation structure in the BP regression model, Famoye [37] introduced a BNB regression model to analyze bivariate data, addressing over-dispersion in it. This model can be used to have a negative, zero, or positive correlation. It uses separate dispersion parameters for each marginal distribution, which can be shown as follows:

$$\mathrm{p}\left(\mathrm{X},\mathrm{Y}\right)=\left\{\prod_{\mathrm{k}=1}^{2}\left(\begin{array}{c}{\mathrm{m}}_{\mathrm{k}}^{-1}+{\mathrm{y}}_{\mathrm{k}}-1\\ {\mathrm{y}}_{\mathrm{k}}\end{array}\right){\left(\frac{{\upmu }_{\mathrm{k}}}{{\mathrm{m}}_{\mathrm{k}}^{-1}+{\upmu }_{\mathrm{k}}} \right) }^{{\mathrm{y}}_{\mathrm{k}}}{\left(\frac{{\mathrm{m}}_{\mathrm{k}}^{-1}}{{\mathrm{m}}_{\mathrm{k}}^{-1}+{\upmu }_{\mathrm{k}}}\right) }^{{\mathrm{m}}_{\mathrm{k}}^{-1}}\right\}\times \mathrm{p }(\mathrm{X},\mathrm{Y})=\left\{\prod_{\mathrm{k}=1}^{2}\left(\begin{array}{c}{\mathrm{m}}_{\mathrm{k}}^{-1}+{\mathrm{y}}_{\mathrm{k}}-1\\ {\mathrm{y}}_{\mathrm{k}}\end{array}\right){\left(\frac{{\upmu }_{\mathrm{k}}}{{\mathrm{m}}_{\mathrm{k}}^{-1}+{\upmu }_{\mathrm{k}}} \right) }^{{\mathrm{y}}_{\mathrm{k}}}{\left(\frac{{\mathrm{m}}_{\mathrm{k}}^{-1}}{{\mathrm{m}}_{\mathrm{k}}^{-1}+{\upmu }_{\mathrm{k}}}\right) }^{{\mathrm{m}}_{\mathrm{k}}^{-1}}\right\}\times \left\{1+\uplambda ({\mathrm{e}}^{-{\mathrm{y}}_{1}}-{\mathrm{c}}_{1})({\mathrm{e}}^{-{\mathrm{y}}_{2}}-{\mathrm{c}}_{2})\right\}$$

$${\mathrm{c}}_{\mathrm{k}}={\left\{(1-{\uptheta }_{\mathrm{k}})/(1-{\uptheta }_{\mathrm{k}}{\mathrm{e}}^{-1})\right\}}^{{\mathrm{m}}_{\mathrm{k}}^{-1}} {\uptheta }_{\mathrm{k}}={\upmu }_{\mathrm{k}}/({\mathrm{m}}_{\mathrm{k}}^{-1}+{\upmu }_{\mathrm{k}})$$

$$\mathrm{k}=\mathrm{1,2}$$

That ${\mathrm{m}}_{\mathrm{k}}$ is the dispersion parameter for the negative binomial distribution.

### Dirichlet negative multinomial regression

DNM distribution [38] is a discrete multivariate distribution that supports the count variables. Suppose $({\mathrm{Y}}_{1},\dots ,{\mathrm{Y}}_{\mathrm{m}})$ are independent Poisson variables with mean vector (${\uplambda }_{1}\mathrm{b},{\uplambda }_{2}\mathrm{b},\dots ,{\uplambda }_{\mathrm{m}}\mathrm{b})$. If gamma (${\mathrm{Y}}_{0},{\mathrm{Y}}_{0})$ that ${\mathrm{Y}}_{0}$ is a shape parameter. If ${\uplambda }_{\mathrm{i}}$ are fixed and known, then $({\mathrm{Y}}_{1},\dots ,{\mathrm{Y}}_{\mathrm{m}})$ has a NM distribution and if ${\uplambda }_{\mathrm{i}}$ is positive random variable, it has the DNM distribution. Mass function for DNM distribution with parameters ${\mathrm{Y}}_{0}, {\uplambda }_{1},\dots ,{\uplambda }_{\mathrm{m}}$ is

$$\frac{\Gamma ({\mathrm{Y}}_{*})}{\Gamma ({\mathrm{Y}}_{0})\prod_{\mathrm{j}=0}^{\mathrm{m}}\Gamma ({\mathrm{Y}}_{\mathrm{j}+1})}\times \frac{\Gamma ({\uplambda }_{8})}{\prod_{\mathrm{j}=1}^{\mathrm{m}}{\uplambda }_{\mathrm{j}}}\times \frac{\prod_{\mathrm{j}=0}^{\mathrm{m}}\Gamma ({\mathrm{Y}}_{\mathrm{j}}+{\uplambda }_{\mathrm{j}})}{\Gamma ({\mathrm{Y}}_{*}+{\uplambda }_{*})}$$

$${\mathrm{Y}}_{*}={\mathrm{Y}}_{0}+\dots +{\mathrm{Y}}_{\mathrm{m}}$$

$${\uplambda }_{*}={\uplambda }_{1}+\dots +{\uplambda }_{3}$$

### Diagonal inflated bivariate poisson regression

The DIBP regression [27] is modified of BP regression that addressed over-dispersion and under-dispersion. Under the PB regression model, the DIBP model is defined as follows:

$${\mathrm{f}}_{\mathrm{IBP}}\left(\mathrm{X},\mathrm{Y}\right)=\left\{\begin{array}{c}\left(1-\mathrm{p}\right){\mathrm{f}}_{\mathrm{BP}}\left(\mathrm{X},\mathrm{Y}|{\uplambda }_{1},{\uplambda }_{2},{\uplambda }_{3}\right) X\ne Y\\ \left(1-\mathrm{p}\right){\mathrm{f}}_{\mathrm{BP}}\left(\mathrm{X},\mathrm{Y}|{\uplambda }_{1},{\uplambda }_{2},{\uplambda }_{3}\right)+p{\mathrm{f}}_{\mathrm{D}}\left(\mathrm{X}|\mathrm{D}\right) X=Y\end{array}\right.$$

${\mathrm{f}}_{\mathrm{D}}\left(\mathrm{X}|\mathrm{D}\right)$ is discrete probability distribution that proper choice for it, is the Poisson or geometric distribution.

### Comparing regression models

Mean Square Error (MSE) is a measure of the error model by average squared differences between the estimated response value from the fitted model and observed value. If the fitted model is acceptable, the observed and their estimated values will be close and MSE to be small. Besides, Akaike Information Criterion (AIC) index that measures the basis of log-likelihood is used for comparing model and smaller values to be well.

### Overdispersion

OV is one of the most important features of counting data. To investigate the OV in univariate analysis, z test was used in which it is assumed that the variance of a variable is $\mathrm{var}\left(\mathrm{Y}\right)=\upmu +\mathrm{c}\times \mathrm{f}(\upmu )$ that if c is zero, there is no OV. Furthermore, for bivariate analyzes, OV will be evaluated based on the chi-square test [38, 39].

## Results

### Frequency of injuries and death

**Fig. 2:**
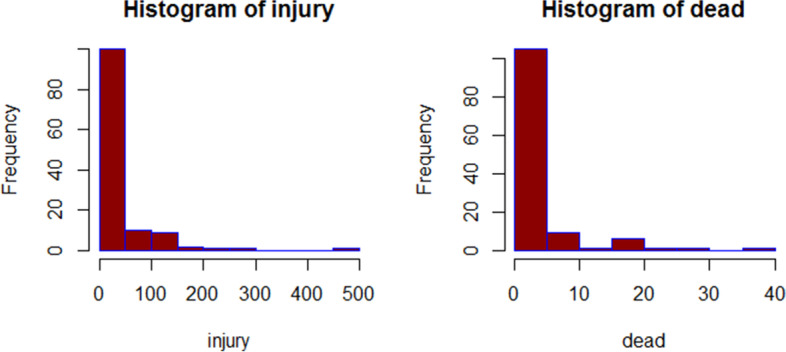
Histogram of the frequency of injuries and fatalities accidents on the roads of Kermanshah in march2020 to march2021

**Fig. 3:**
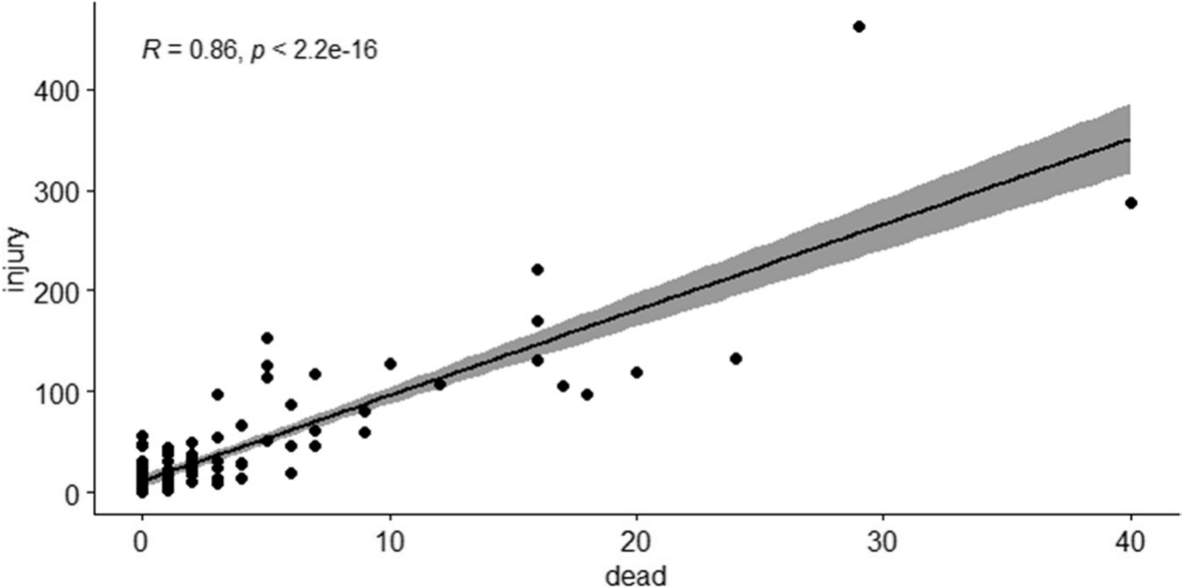
Correlation between the number of fatalities and injuries in road accidents in Kermanshah in march2020 to march2021

Based on density characteristics, accidents were split into 125 regions. The distribution of the number of injuries and fatal accidents was observed using a histogram plot (Fig. 2). The data is not symmetrically distributed and is biased to the right, as can be observed. The number of wounded in each location is 38.41(60.034) individuals on average (SD), with a median of 18.5 people. Moreover, the mean (SD) for the number of fatal in each area is 3.5(5.91) people with a median of 1 person. The correlation between the number of injuries and deaths in terms of road accidents was 0.856 and shows that these two response variables positively correlate with each other, shown in Fig. 3. This research looked at seventeen factors, including the frequency of accidents, accident states, and accident status. Overturning and collisions of cars with each other or multiple vehicles resulted in the most accidents, whereas collisions with a stationary object resulted in the fewest. Most of the vehicles collided were happened in Kamyaran route, which also has the highest number of injuries. The highest number of deaths is related to the accident of several vehicles, the most dangerous type of which is a face-to-face collision on non-separate two-way axes, antenna intersections, and non-standard U-turns.

### Evaluate overdispersion

As shown in Table 2, the OV test was significant for both response variables (death and injury) in univariate analysis and injury variables in bivariate analysis.

**Table 2:** Test for over-dispersion in univariate and bivariate regression models

Outcomes	Univariate			Bivariate	
	Z	alpha	*p*-value	Chi-square	*p*-value
death	2.64	0.746	0.004	59.649	0.0078
injured	2.013	0.632	0.001	13.284	0.9998

There was significant OV in death and injury univariate model and in bivariate death model.

### Fitted count regression models

#### Univariate count models

First, results consider and compare two common univariate models (P regression and NB regression) for the number of injuries and deaths.

P regression and NB regression were used to investigate the relationship between the number of injured with type and cause of the accident. As can be seen in Table 3, the results of NB regression and P regression are different due to the overdispersion feature of the data and therefore the NB regression gives more reliable results. The results show that the multi-vehicle collision, riding with riding, overturning, and pedestrian and riding a motorcycle had a significant relationship with the number of injured in the accident. P regression and NB regression were also used to investigate the relationship between the number of death and the type and shape of the accident. In regression models, multi-vehicle collision, ride-on-ride, pedestrian and motor-ride had a significant relationship with the number of death.

**Table 3:** Univariate regression count model: parameter estimation for injuries and deaths

Factor	Univariate Regression			
	P		NB	
	Death	Injured	Death	Injured
**Accident number**	0.0485 (0.0451)	-0.0696 (0.0134)^a^	0.0485 (0.0451)	-0.0792 (0.0447)
**Fixed objects**	-0.0172 (0.0857)	0.1838 (0.0262)^a^	-0.0172 (0.0857)	0.2132 (0.0813)^a^
**Multiple-vehicle collision**	0.1623 (0.0965)	0.3745 (0.0290)^a^	0.1623 (0.0965)	0.3575 (0.0928)^a^
**Head-On Collision**	-0.0565 (0.0606)	0.0978 (0.0183)^a^	-0.0565 (0.0606)	0.0348 (0.0678)
**Truck collision**	-0.0605 (0.0734)	0.0699 (0.0221)^a^	-0.0605 (0.0733)	0.1241 (0.0697)
**vehicle collision**	-0.0194 (0.0525)	0.1096 (0.0165)^a^	-0.0194 (0.0525)	0.1221 (0.0520)^a^
**Motorcycle collision**	-0.0264 (0.0582)	0.1614 (0.0177)^a^	-0.0264 (0.0582)	0.1747 (0.0569)^a^
**Pedestrian**	-0.1912 (0.0705)^a^	-0.0034 (0.0199)	-0.1912 (0.0705)^a^	0.0461 (0.0623)
**Vehicle Rollover**	-0.0424 (0.0445)	0.1217 (0.0128)^a^	-0.0424 (0.0445)	0.1293 (0.0435)^a^
**Other type of accidents**	0.0195 (0.0620)	0.1063 (0.0173)^a^	0.0195 (0.0620)	0.1194 (0.0620)
**turn left without using the turn signal**	0.0413 (0.0244)	-0.0104 (0.0077)	0.0414 (0.0245)	-0.0122 (0.0291)
**Over-speeding**	-0.0081 (0.0317)	-0.0387 (0.0091)^a^	-0.0816 (0.0317)	-0.0152 (0.0272)
**Passing without signaling**	-0.0495 (0.0787)	-0.0652 (0.0249)^a^	-0.0496 (0.0787)	-0.1660 (0.0843)^a^
**Lack of attention to the front**	0.1044 (0.0259)^a^	0.0195 (0.0081)^a^	0.1044 (0.0259)^a^	-0.0092 (0.0266)
**neglect of the right of priority**	0.1589 (0.0498)^a^	0.0195 (0.049)	0.1588 (0.0498)^a^	0.0332 (0.0491)
**transverse distance**	-0.1391 (0.1275)	-0.1055 (0.0367)^a^	-0.1391 (0.1275)	-0.1807 (0.113)
**Breaking traffic rules**	-0.1966 (0.0611)^a^	-0.1417 (0.017)^a^	-0.1967 (0.0612)^a^	-0.1058 (0.0593)

^a^ Significant at 0.05 level

#### Bivariate count models

This paper considers and compares four different candidate bivariate models (BP regression, BNB regression, DIBP regression, and DNM models) for the number of injuries and deaths. Results indicated that the most of predictors are significant under Poisson regression model for injury however some of predictors was found significant using bivariate count regression model so the results of univariate and bivariate models are different. Deviance to the left and colliding with a pedestrian were two factors that became significant in both death and injury models (Table 4).

**Table 4:** bivariate count regression modes:l parameter estimation for injuries and deaths

Factor	Bivariate Regression								
	BP		BNB		IDBP		DNM		
	Death	Injured	Death	Injured	Death	Injured	Death	Injured	Wald
**Accident number**	0.089 (0.048)	0.1195 (0.0129)^a^	0.2327 (0.1825)	1.0879 (0.5946)	0.4777 (0.1179)	-0.1385 (0.4224)	0.0468	0.3986	2.653
**Fixed objects**	0.0535 (0.0916)	-0.1666 (0.0259)^a^	-0.4258 (0.3297)	-0.2979 (1.0742)	0.2615 (0.1168)	0.3861 (0.2872)	0.6478	0.5948	0.9878
**Multiple-vehicle collision**	0.2588 (0.1030)^a^	0.0433 (0.0273)	0.2596 (0.3807)	-1.1074 (1.2403)	0.0202 (0.1567)	0.1491 (0.1116)	0.6759	0.8604	2.2006
**Head-On Collision**	0.0886 (0.0637)	0.1677 (0.0173)^a^	0.2323 (0.2789)	0.2515 (0.9089)	-0.7134 (0.3658)	0.6766 (0.1839)	-0.7321	0.8818	3.9526
**Truck collision**	-0.0881 (0.0796)	-0.1365 (0.0224)^a^	0.0076 (0.2854)	1.6612 (0.9298)	-0.3457 (0.2889)	0.2850 (0.0456)	0.0609	1.0563	3.4932
**vehicle collision**	0.0619 (0.0553)	0.1577 (0.0158)^a^	-0.0567 (0.2116)	1.9764 (0.6894)^a^	-0.3288 (0.1263)	0.3495 (0.3348)^a^	-0.5637	0.4786	2.8534^a^
**Motorcycle collision**	0.035 (0.0617)	0.1799 (0.0172)^a^	0.3681 (0.2309)	0.5088 (0.7525)	0.8051 (0.4824)^a^	0.3131 (0.5022)	0.3233	0.1914	2.9601
**Pedestrian**	0.256 (0.0763)^a^	0.2041 (0.0197)^a^	0.6339 (0.2518)^a^	1.6878 (0.8206)	-0.8117 (0.0716)	-0.7549 (0.369)	0.7327	0.6152	3.4130^a^
**Vehicle Rollover**	0.0629 (0.0474)	0.1335 (0.0116)^a^	0.2855 (0.1775)	0.8541 (0.5784)	0.6751 (0.0545)	0.386 (0.4372)	0.4288	0.3233	4.5605
**Other type of accidents**	0.0039 (0.0656)	0.0526 (0.0165)^a^	0.085 (0.2551)	0.5196 (0.8312)	-0.6574 (0.2267)	0.6502 (0.4032)	0.0471	0.0781	0.1137
**turn left without using the turn signal**	0.0476 (0.0255)	0.0796 (0.0077)^a^	0.3664 (0.1212)^a^	1.2507 (0.3948)^a^	0.8269 (0.2394)^a^	0.7106 (0.2114)^a^	0.1975	0.1424	0.3086^a^
**Over-speeding**	0.0409 (0.0336)	0.0247 (0.0093)^a^	0.1165 (0.1098)	0.2509 (0.358)	0.9842 (0.0294)	0.3647 (0.1418)	0.2769	0.3049	1.8007
**Passing without signaling**	-0.8884 (0.0842)	0.0067 (0.0240)	-0.0233 (0.358)	1.1216 (1.1664)	-0.5923 (0.1172)	0.6367 (0.2496)	0.1502	0.2328	0.3381
**Lack of attention to the front**	0.1324 (0.0272)^a^	0.099 (0.0077)^a^	0.3196 (0.1092)^a^	0.6132 (0.3559)	-0.1372 (0.0598)	0.7405 (0.1345)	0.2407	0.1499	7.7306
**neglect of the right of priority**	-0.1869 (0.0530)	-0.1832 (0.0148)^a^	0.6628 (0.1991)^a^	0.9063 (0.6489)	-0.0981 (0.0522)	0.9309 (0.1579)^a^	0.1845	0.3203	3.3667^a^
**transverse distance**	-0.0768 (0.135)	0.1077 (0.0344)	0.4208 (0.458)	-0.3148 (1.4921)	0.5975 (0.1079)	0.1234 (0.1693)	0.0854	1.0061	1.3972
**Breaking traffic rules**	-0.2212 (0.0641)^a^	0.0623 (0.0174)^a^	0.2463 (0.2395)	1.8660 (0.7805)	0.5125 (0.4309)	0.3545 (0.0066)	0.3188	0.4517	2.4747

^a^ Significant at 0.05 level

#### Comparison of models

In bivariate models, except for the DNM model, there is a reasonable decrease in the AIC measures of the saturated model compared to the reduced model for the other three models. Moreover, the results of MSE suggest that the DIBP model is a better fit for these data. As shown in Table 5 for the injuries model, MSE is lowest, respectively for DIBP (137.87), BNB (289.46), BP (412.36) and DNM (3640.89) models. These results are established for dead models.

**Table 5:** Goodness of fit test statistics for univariate and bivariate count regression models

Index		Univariate Regression			Bivariate Regression		
		P	NB	BP	BNB	IDBP	DNM
**MSE**	Injury death	0.258	0.267	412.36	289.46	137.87	3640.89
		0.261	0.261	12.16	4.012	1.71	36.62
**AIC**	Fitted model	411.59^a^	413.59^a^	1402.48	963.2	1625	397.45
	Reduced model	603.63^a^	2768.1^a^	1897.54	986.94	2689	398.34

^a^ For death model

## Discussion

Forecasting based on facts, accessible parameters, and available information are the major aims of modeling and categorization in statistics, and there are numerous statistical approaches for achieving these goals and modeling. Because of the rising number of road accidents in the nation, having an indicator that displays the current state of road safety may be required for controlling road traffic accidents, thus study in this area is critical. Most fatalities in traffic accidents are caused by dangerous drivers and people living in low- and middle-income countries, where transportation is increasing. Many reasons, such as rapid urbanization, poor road safety, poor law enforcement, distracted or tired driving, drug or alcohol use, speeding, and not wearing seat belts and helmets, contribute to traffic accidents [40]. This data was shown that 45% of accidents were due to speeding, 23.33% due to left turn and 10.83% due to neglect of the right of priority. Also, the condition of the roads also affects the occurrence of traffic accidents. About 50% of the dead and 37% of the injured in the province occurred in accidents of 9 main routes, of which five routes are non-separate and narrow. Two axes are widening whenever their completion is delayed, it is directly effective in increasing the number of deaths and injuries in the province. The two alternative routes are national and international highways, which must protect the remaining 15 kilometers between Kermanshah and Islamabad, as well as expand the number of roadside resorts and electronic control systems. The data showed that 45% of accidents were due to speeding, 23.33% due to left turn, 10.83% due to non-compliance with the priority.

Identifying the factors affecting deaths and injuries from road accidents is essential in health system policy-making in reducing mortality. It is necessary to use statistical analysis, such as the count nonlinear regression model to better describe and analyze the number of accident data and find the impact of humans, road conditions, and vehicle type on traffic accidents. On the other hand, road accidents have consequences such as death and injury, and the simultaneous analysis and description of these two responses have rarely been used in studies. Therefore, the purpose of this study is to compare the fit of several bivariate regression models applied to road accident data and compare them. BP model is the most widely used model for two-variable counting. However, as demonstrated in the findings, the univariate Poisson model's limitations apply here as well, and model variance estimations are influenced by over- or under-dispersion in the data. Furthermore, the presence of negative correlation and heterogeneity in variance diminishes the model's effectiveness. The BNB regression model is used to represent paired counting data with interdependent response variables and the OV feature [18]. But, the issue of negative correlation still applies here. Therefore, a major drawback of the above models is their ability to model data only with a positive correlation. Besides, because they are Poisson marginal distributions, they cannot model over-scattering or low-scattering. In the case of sparse count data, Poisson mixed models are potentially useful, and some other models make negative correlations possible. However, such models involve difficult and complex calculations to estimate. Another model is the IDBP model, which is computationally feasible and allows for OV and negative correlation. The DNM model is a good marginal regression model for counting data that takes into account differences between and within units. As a result, if a marginal model is sought, the DNM model offers an appropriate structure. In particular, having separate mean parameters for each component and two variance parameters makes this model suitable with unbalanced panel counting data with a stable covariance structure.

Our results show the suitability of the IDBP model for analyzing accident datasets. In the IDBP death model, the variables of a motorcycle accident, pedestrian accident, left turn deviance, and unsafe speeding was significant, and these factors were related to accident mortality. In IDBP injury model, the variables of a driving accident, pedestrian accident, left turn deviance, and non-observance of the right of priority was significant, and these factors were related to injury in the accident. Major pedestrian accidents are in terms of the compulsion of pedestrians in traffic from the environment or a place originally designed for using vehicles. Unfortunately, the high number of pedestrian accidents in the country and the number of casualties and disabilities caused by it have created many obvious and hidden problems for society. Overtaking and veering to the left are the province's most dangerous driving violations. Overtaking violations and left-hand deviations occur on non-separate two-way roadways that result in head-on collisions. For this reason, seven axes of the province are vital, and as long as their return routes are not separated, this sort of violation and the consequent losses will occur.

The findings of the present study are consistent with the results of Al-Ghamdi's study of traffic accident data. In this study, logistic regression was used to examine the contribution of several variables in the severity of the accident. Out of 9 independent variables obtained from police accident reports, two variables; the location and cause of the accident were significantly related to the severity of the accident [41]. Abdissa Aga et.al used univariate count regression models to analysis the factors associated with the number of human deaths from road traffic accidents. The study aimed to identify the potential factors associated with the number of human deaths by road traffic accidents in the Oromia Regional State, Ethiopia. The hurdle Poisson regression model was shown most appropriate model from other common count models. The results are shown that the number of deaths due to driving in an illegal way compared to drivers denying priority to pedestrians was lower [42].

Unfortunately the number of studies that compared bivariate count models is very small. The results obtained from comparison of models in this study are consistent the study of Famoye in 2010 which showed that BNB regression model performs better than the BP regression model [43]. Rafiqul et.al, introduced a bivariate zero-truncated Poisson regression model based on a conditional model for accident data. Two correlated outcome variables were frequency of cars and number of casualties in accidents. Based on goodness of fit index , AIC, BIC and deviance, results are shown that the proposed full model provides the best fit [44].

## Conclusions

The data show that amending in legislation, vehicle standards and access to post-accident care has been developed. However, this progress has not been rapid enough to offset the consequences of traffic accidents that occur in many parts of the world specially developing country [40].

The objective of this study was to evaluate the statistical model of road accidents in Iran. The P and NB regression model was used for univariate fitting the relationship between road accidents injuries and fatalities and their contributing factors which are reckless driving, careless pedestrians, high speed, defective motor vehicles, motor cyclists and other factors including slippery road and poor visibility. The OV test was carried out and it was shown, there was over-dispersion in the data and so NB regression was more reliable. However because there were 2 correlated count responses (injury and death), bivariate count models was used to improve results. Finally, basis on GF test and MSE index, IDBP model is a better fit for this data and vehicle collision, turn left without using signal and neglect of the right of priority for injured response and motorcycle collision and turn left without using signal for dead response are significant.

## Supplementary Information

**Additional file 1.**

**Additional file 2.**

**Additional file 3.**

## Data Availability

All data generated or analyzed during this study are included in this published article.

## Abbreviations

P: Poisson
NB: Negative Binomial
BP: Bivariate poisson
BNN: Bivariate negative binomial
DNM: Dirichlet negative multinomial
DIBP: Diagonal inflated bivariate Poisson
MSE: Mean Square Error
AIC: Akaike Information Criterion
OV: Overdispersion
